# Physicians’ Complex Relationships With Health Information Technology and Burnout: Cross-Sectional Survey Study

**DOI:** 10.2196/93266

**Published:** 2026-09-23

**Authors:** Mavis Jones, Timothy Jason, Dara Liu, Jeannette Comeau, Chandi Chandrasena, Noni E MacDonald, Janice E Graham

**Affiliations:** 1 OntarioMD, 150 Bloor Street, Toronto, ON, M5S 2Y5, Canada; 2 Department of Pediatrics, Dalhousie University, 5849 University Avenue, Halifax, NS, B3H 4H7, Canada, 1 9024941897

**Keywords:** burnout, digital health, EMR, physician wellness, stress, health care workforce, administrative burden, electronic medical record

## Abstract

**Background:**

Health information technology (HIT), while designed to improve practice efficiency and patient care, can contribute to physician burnout when designed without clinical practice in mind. Administrative and clinical demands now require physicians to spend more time on HIT, a burden that contributes to burnout and affects time with patients.

**Objective:**

This study had two objectives: (1) to examine how physician perceptions of specific HIT functions may alleviate or contribute to physician burnout within the differing health care landscapes of Ontario and Nova Scotia, with particular attention given to physician perceptions of HIT associated with administrative burdens, interoperability, and system integration, and (2) to explore physician experiences and perceptions of HIT as potential factors affecting physician burnout, with the goal of delivering findings (eg, a model) that could be applied to improve physicians’ HIT experience.

**Methods:**

We designed an exploratory mixed methods study that deployed a cross-sectional survey in 2 Canadian provinces: Ontario and Nova Scotia. Centralized clinician management software (Ontario) and medical association networks (Nova Scotia) were used to recruit from an estimated 35,341 (Ontario) and 2809 (Nova Scotia) physicians. The survey was distributed between February and April 2024. Nonphysician clinicians, clinic staff, and non-HIT users were excluded, resulting in 1245 Ontario and 136 Nova Scotia physician HIT-user respondents. For both the Ontario and Nova Scotia samples, descriptive analyses of quantitative survey items were compared, and open-text responses were subjected to qualitative coding for themes. Subgroup differences in HIT-related burnout were analyzed in the larger Ontario sample.

**Results:**

Common experiences were apparent despite differences in the samples, provincial health systems, and available HIT. “Managing communications related to patient care” and “inputting data into your EMR” were among the top 3 administrative burdens. While “logging in and out of technology platforms” ranked higher in Nova Scotia as an administrative burden, the related theme of integration and interoperability was prominent in both samples. In the Ontario sample, perceptions of HIT, quality of support, and hours worked per week accounted for over half of the variation in self-reported burnout.

**Conclusions:**

While physicians appreciate the advantages of HIT for patient care, they also experience an overwhelming administrative and documentation burden, as well as disjointedness across data platforms, which contributes to their burnout. Greater ongoing involvement by end users in the design and usability of these technologies, along with improved standardization and interoperability, would reduce these burdens while maintaining the benefits of digital health systems.

## Introduction

Physician burnout remains a system-wide problem with consequences for physicians and health care delivery in Canada. The Canadian Medical Association’s (CMA) 2025 National Physician Health Survey found that 46% of physicians (n=3310) reported symptoms of burnout [1] (high levels of at least one indicator of depersonalization or emotional exhaustion using the Maslach Burnout Inventory [2]). The CMA study connects burnout and related health outcomes to an escalation in administrative burden: over the 3 iterations of their survey—2017, 2021, and 2025—there has been a significant increase in the proportion of respondents reporting spending an excessive or moderately high amount of time on their electronic medical record (EMR) on administrative tasks outside of regular hours, from 36% to 49% in 2021 and 64% in 2025. Here and elsewhere, it has been found that the burden is particularly high in family medicine [3]. One study reported that this group spends an average of 19 hours a week on administrative tasks [4]. This has an impact on access to primary care when family doctors experience administrative demands competing for patient-facing time and/or reduce (or leave) clinical practice due to burnout.

While workload, administrative burdens, and insufficient time with patients are neknown contributors to physician burnout [5,6], health information technology (HIT)—encompassing the electronic systems designed to collect, share, and manage patient information—has introduced new dynamics [7-9]. Paradoxically, the technological solutions intended to support a physician’s daily work can also add to their workday burden, as they adapt to a tsunami of expensive, new, and largely unregulated HIT platforms while having a minimal role in their design and implementation [10,11].

**Figure 1.**
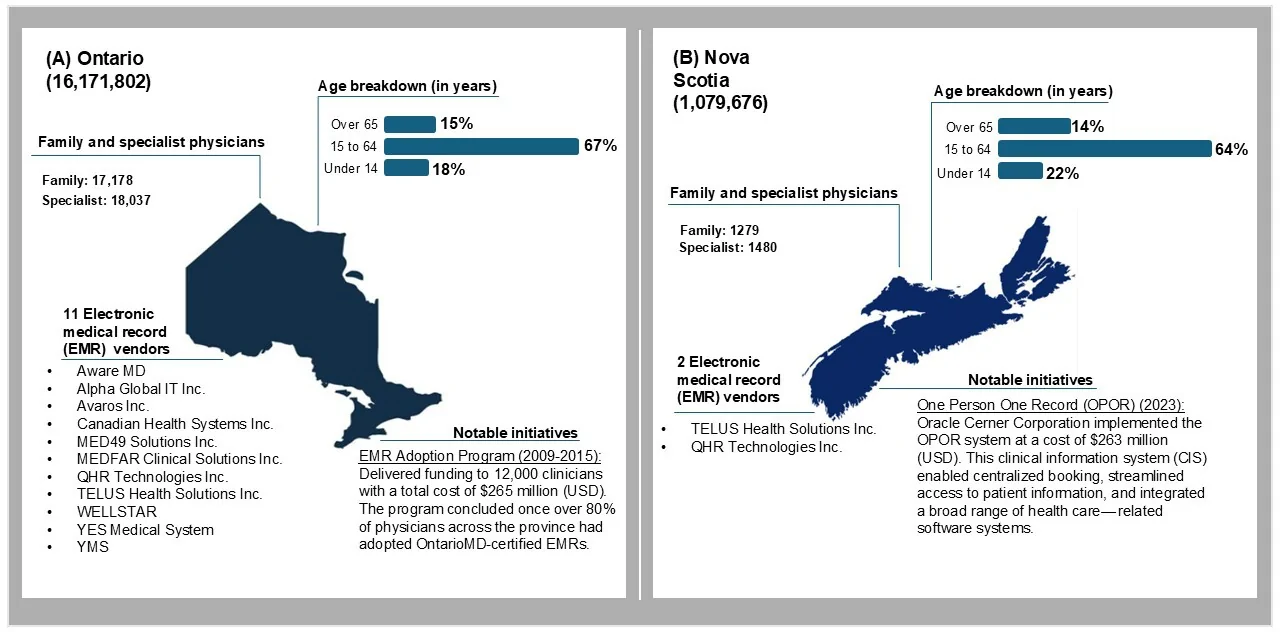
Comparative context. (A) Ontario and (B) Nova Scotia.

In Canada, while federal initiatives shape the overall approach to health nationwide, provinces are responsible for (and fund) health care delivery. This leads to differences in models for care delivery that can affect physician workload—for example, in Ontario, a primary care physician in a Family Health Team may have access to more resources than a primary care physician working outside that model. Provinces are also responsible for decisions affecting the technologies clinicians use to manage patient data and to interact with others in the provincial health system, including the implementation of (provincial) system-wide initiatives. Our study focuses on 2 settings—the Canadian provinces of Ontario and Nova Scotia—to explore physicians’ HIT experiences, including potential contributors to burnout in health care providers, their use of HIT, and notable HIT initiatives (Figure 1) [12-16].

While previously relying on 1 of 2 EMRs on the market in the province, Nova Scotia physicians only recently (December 6, 2025) began the implementation of One Person One Record (OPOR) [17], a clinical information system designed by Oracle Cerner to address centralized booking, access to patient information, and integration across institutions and software systems. In contrast, community-based Ontario physicians have had greater choices available to them via certification and adoption programs beginning in 2009 [18], allowing physicians to choose from a list of certified offerings meeting a minimum standard. Physicians working in both community and hospital settings rely on hospital information systems (commonly referred to as HIS) such as EPIC. Both provinces feature multiple platforms for transmitting reports from hospitals, laboratories, and diagnostic facilities to a patient’s electronic record. In addition, physicians may have access to add-ons, only some of which may be integrated (ie, accessible directly from the EMR without having to log out and log in to a different platform with a different username and password).

The relative maturity of the HIT landscape in Ontario is a consequence of provincial government investment, which includes change management support via a team of field advisors and peer support (ie, physicians and other clinical staff) who connect physicians with resources, provide guidance, and offer other change management support for adopting and implementing new technologies (physicians are free to choose a noncertified EMR outside of this system). Government-funded programs are delivered by OntarioMD (OMD, a subsidiary of the Ontario Medical Association). The closest analog to OMD in Nova Scotia would be the E-Health division of the provincial medical association, Doctors Nova Scotia, which provides physician support, advice, and guidance on these technologies.

We designed a mixed methods study to explore physicians’ perceptions of HIT and their relationship to their burnout. As described, Ontario and Nova Scotia are not directly comparable, given their differences in size, HIT infrastructure, and historical support for HIT adoption. We hypothesized that, despite these differences in maturity, we may see commonalities across Ontario and Nova Scotia physicians in terms of the benefit they derive from HIT, their expectations vs the reality of their experience, their usability challenges, and the ways in which the day-to-day world of clinical practice is affected by HIT. Through this exploratory, contextual analysis, we sought to learn which aspects of HIT are perceived by physicians as valuable and which they perceive as contributing to their burnout; the balance between the 2 shapes their overall experience. Our secondary objective, given sufficient data, was to run tests of significance, which could contribute to a model for understanding the factors that contribute to tech-related burnout.

## Methods

### Survey Instrument

Between 2018 and 2024, OMD conducted an annual survey of clinicians as an evaluative mechanism (internal purposes only) to provide insight into the impact of physician-facing initiatives and to inform future priorities. Within this survey, there was a section on challenges, with optional open-text questions that were reviewed and coded for themes. The cumulative findings suggested that a focused interrogation of the impact of technology on physicians was warranted.

We designed and conducted a self-administered electronic questionnaire on HIT-related burnout, including potential factors, guided by (1) a literature review on HIT-related factors contributing to physician burnout, (2) a review of qualitative responses to annual OMD surveys on challenges with HIT, and (3) team discussions of evidence to date. The questionnaire was piloted by the Nova Scotia E-Health Committee, comprising 9 physicians and 2 medical learners, and then refined with support from OMD-affiliated physicians. The final 25-item instrument consisted of 23 mandatory closed-ended and 2 open-ended questions. The first question asked whether respondents were currently using HIT in their primary practice; those who responded “no” were excluded from subsequent questions on HIT. Three potentially sensitive questions in the “About You” section included “prefer not to answer” as an option. Two versions containing the same content specified regional consent and identification details (Multimedia Appendix 1). Microsoft Dynamics 365 Customer Voice was used to develop and distribute both questionnaires separately via links embedded in email and on social media. Respondents could complete the questionnaire at their own pace and edit previous responses, if necessary. No incentives were provided for survey completion. Survey design is reported according to the CHERRIES checklist [19].

### Sampling and Distribution

The questionnaire was launched on February 14, 2024, with an in-field period until April 5, 2024. In Nova Scotia, distribution used nonprobability voluntary response sampling [20] via Dalhousie Faculty of Medicine (FoM) communications, the website, social media, and department newsletters. The Doctors Nova Scotia e-newsletter and discussion forum and the *Nova Scotia Health (NSH*) newsletter also distributed the e-questionnaire and reminders. In Ontario, the questionnaire was distributed using OMD’s customer relationship management software to an estimated sampling frame of between 24,000 and 31,000 Ontario physicians and other clinical staff. Reminder emails to complete the questionnaire were sent at regular intervals. Additionally, social media posts were shared throughout the distribution period on behalf of both OMD and the Ontario Medical Association. Unique identifiers in the form of College of Physicians and Surgeons of Ontario registration numbers precluded duplicate responses. Eligibility criteria of “current use of HIT” and “family physician” or “specialist” were applied to ensure a valid sample of respondents according to study objectives.

### Analysis

#### Quantitative

Using Microsoft Power Query and Power BI, Ontario and Nova Scotia data were cleaned and organized separately due to the qualitative and methodological distinctiveness of the 2 samples, thereby addressing potential methodological, statistical, and interpretive errors [21]. Structured data models and tables were derived using step-by-step queries for export into the R Studio environment (version 2024.04.2+764 [2024.04.2+764]; Posit Software, PBC). After consulting the relevant literature [22,23], we determined that there was insufficient power in the Nova Scotia sample for significance testing due to the bias inherent in small sample sizes. We then limited our comparative analysis to descriptive data. For numeric data, measures of central tendency (mean) and dispersion (SD and range) were calculated. For categorical data, frequency counts or percentages were calculated.

Statistical tests of reliability and internal item consistency were conducted on the Ontario perceptions scale data to evaluate psychometric properties, as well as the potential to calculate an overall score on perceptions. The perceptions scale data showed a standardized Cronbach α criterion of 0.854 and moderate intercorrelations (Table 1). A polychoric factor analysis with equamax rotation yielded a 2-factor solution, which could be described as *positive perceptions about HIT* (ie, “easier,” “communication,” “revolutionize,” “access data,” “quality care,” and “patient coordination”) and *negative perceptions about HIT* (ie, “negative impact,” “reason for job stress,” and “prevent patient care”). To derive an overall numeric score for perceptions as a proxy measure of attitude toward HIT, each item’s Likert-scale categories were converted into numeric equivalents, with scores of 1 for “Strongly Disagree” and 5 for “Strongly Agree.” The 3 *negative perceptions about HIT* items were reverse scored. The resulting overall perception score ranged from 9 to 45, with higher scores indicating more favorable attitudes toward HIT.

**Table 1.** Intercorrelations matrix for perceptions scale items.

	Access data	Communication	Patient coordination	Easier	Negative impact	Prevent patient care	Quality care	Reason for job stress	Revolutionize
Access data	—	0.380^a^	0.396^a^	0.341^a^	–0.287^a^	–0.191^a^	0.383^a^	–0.223^a^	0.266^a^
Communication	0.380^a^	—	0.397^a^	0.399^a^	–0.372^a^	–0.238^a^	0.398^a^	–0.321^a^	0.309^a^
Patient coordination	0.396^a^	0.397^a^	—	0.426^a^	–0.368^a^	–0.226^a^	0.503^a^	–0.259^a^	0.298^a^
Easier	0.341^a^	0.399^a^	0.426^a^	—	–0.545^a^	–0.425^a^	0.542^a^	–0.505^a^	0.430^a^
Negative impact	–0.287^a^	–0.372^a^	–0.368^a^	–0.545^a^	—	0.601^a^	–0.475^a^	0.658^a^	–0.353^a^
Prevent patient care	–0.191^a^	–0.238^a^	–0.226^a^	–0.425^a^	0.601^a^	—	–0.332^a^	0.591^a^	–0.217^a^
Quality care	0.383^a^	0.398^a^	0.503^a^	0.542^a^	–0.475^a^	–0.332^a^	—	–0.372^a^	0.455^a^
Reason for job stress	–0.223^a^	–0.321^a^	–0.259^a^	–0.505^a^	0.658^a^	0.591^a^	–0.372^a^	—	–0.264^a^
Revolutionize	0.266^a^	0.309^a^	0.298^a^	0.430^a^	–0.353^a^	–0.217^a^	0.455^a^	–0.264^a^	—

a *P*<.0001.

Ontario numeric data were assessed for evidence of normal distributions and outliers. Outliers were identified by examining boxplots and normal curves, as well as via statistical tests such as the Rosner test. Subject matter experts were consulted to determine plausible upper and lower limits for a range of values. These included the 3 physician coauthors and 2 OMD colleagues with decades of experience working with clinical practices on the use of HIT in its various forms. Outliers identified in the following variables were replaced with the median of the distribution: “hours per week” (6 values); “hours per week on admin” (10 values); “extra hours per week on admin” (8 values); “% of time spent on admin” (4 values); “% on patient care” (1 value); “% burnout from tech” (1 value); “age” (3 values); and “years of practice” (3 values).

Respondents assessed their level of tech-related burnout on a scale of 0 (no burnout) to 10 (extreme burnout). Respondents were asked to rank 10 administrative tasks (validated through a systematic literature review, consultation, and the results of internal qualitative research) in order from greatest to least burden. The percentage of respondents who selected a task as one of the top 3 most burdensome tasks was derived by summing the counts of first, second, and third place responses for each administrative task and then dividing the sums by the number of respondents. Using robust, brute-force, rank-aggregation statistical procedures, the final list of tasks from most to least burdensome was derived.

Due to the type of data and observed nonnormal distributions, nonparametric tests of group differences were selected to better understand tech-related burnout in relation to Ontario sample characteristics. Specifically, tests of group median differences were conducted using Kruskal-Wallis (KW) rank-sum tests for independent samples, as well as follow-up Mann-Whitney *U*-tests, where relevant. To determine statistical associations among numeric data, Pearson product-moment or point-biserial correlation tests were conducted.

Multiple regression analysis was used to identify potential factors associated with self-reported tech-related burnout. For this analysis, tech-related burnout was treated as a continuous numeric outcome variable [24,25]. First, potential factors and covariates were identified. Then, nonparametric tests were conducted systematically for each potential factor or covariate in relation to tech-related burnout to assess statistical significance and effect sizes. Monotonic effects of ordinal-scale variables were assessed using Bayesian generalized nonlinear multilevel modeling methods. Surviving factors were entered systematically into forward and backward stepwise regression tests until the best-fitting parsimonious model was yielded. Due to the large sample size, power, and potential clinical significance of the findings, a stringent alpha criterion of *P*=.001 was used to determine statistical significance.

#### Qualitative

We included open text questions (response required) to collect respondents’ reasoning behind their answers. The main open-text question was “How does health information technology contribute to or help alleviate your burnout?” We reviewed and coded categories and compared emerging themes in the quantitative data and from our literature review on physician burnout [26]. As the responses were in a long-answer format, it was possible to code multiple references within a single response. A total of 43 responses were omitted from the coding report for being either (1) uncodable (eg, a dash, a dot, a question mark, or random letters), (2) some version of “no idea,” or (3) difficult to determine what was meant by the respondent (eg, a phrase that appeared unrelated to the question), even following consultation with other members of the research team. Each team coded separately, led by a coder familiar with their jurisdiction to facilitate interpretation of context-specific references in the open text. Two coders were used for the Ontario sample and 1 for the Nova Scotia sample. While this meant that each team generated its own codebook, our intent was to capture the perspectives of respondents within their regional contexts—given differences in population size, digital maturity, etc—to see whether any areas of commonality emerged despite those differences. After initial coding, codebooks were iteratively refined within the research team, and coding was revisited with discussion where ambiguity or disagreement emerged. Following completion of coding, salient codes were reviewed in detail for common themes across samples.

### Ethical Considerations

The Nova Scotia questionnaire was approved by the Research Ethics Board at Dalhousie University (Halifax, Nova Scotia, Canada, file number: 2023‐6993). As OMD operates under both a provincial government and professional association structure without an institutional ethics board, the Ontario survey was deployed with informed consent language (in consultation with the Dalhousie team and OMD’s legal team) in the survey header, asking participants to read the consent text, which also cites OMD’s privacy policy on information sharing [27]. Participants were asked to confirm they had read the text and that they agreed to participate. Emailed invitations to complete the survey also used language regarding the voluntary nature of participation.

## Results

### Quantitative Findings

Because the objective of the study was to explore *physician* experiences with HIT, 138 Ontario and 20 Nova Scotia nonphysician clinicians and clinic staff, as well as 24 Ontario and 4 Nova Scotia non-HIT physician users, were excluded, resulting in 1245 Ontario and 136 Nova Scotia physician HIT user respondents. Data on clinician and clinical characteristics are reported in Table 2. Significant differences in self-reported tech-related burnout by characteristic are noted for the Ontario sample only.

**Table 2.** Physician and clinical characteristics.

Characteristics	Ontario (N=1245)	Nova Scotia (N=136)
Practice type, n (%)		
Single choice		
Hospital-based	119 (9.6)	66 (48.5)
Group	812 (65.2)	51 (37.5)
Solo	250 (20.1)	12 (8.8)
Other	64 (5.1)	7 (5.1)
Fee structure		
Single choice		
Clinical or academic funding	79 (6.3)	74 (54.4)
Fee-for-service	491 (39.4)	22 (16.2)
Salaried	133 (10.7)	22 (16.2)
Other (eg, capitation models)	542 (43.5)	18 (13.2)
Clinician type^a^		
Single choice		
Family physician	911 (73.2)	53 (39.0)
Specialist	334 (26.8)	83 (61.0)
Gender^b^		
Single choice		
Woman	649 (52.1)	84 (61.8)
Man	559 (44.9)	44 (32.4)
Other	37 (3.0)	8 (5.9)
Age (y), mean (range)	52 (27-79)	50.4 (30-81)
Years of practice (y), mean (range)	22.4 (0-60)	19.5 (1-52)
Racial or ethnic identity, n (%)		
Multiple choice		
East Asian	92 (7.4)	5 (3.7)
South Asian	147 (11.8)	4 (2.9)
Black	21 (1.7)	2 (1.5)
White	744 (59.8)	104 (76.5)
Middle Eastern	95 (7.6)	2 (1.5)
Southeast Asian	13 (1.0)	2 (1.5)
Latin American	15 (1.2)	4 (2.9)
Indigenous	8 (0.6)	—
Prefer not to answer	47 (3.8)	9 (6.6)
Other	63 (5.1)	4 (2.9)
Perceived proficiency using HIT		
Single choice		
High	290 (23.3)	31 (22.8)
Above average	475 (38.2)	43 (31.6)
Average	431 (34.6)	53 (39.0)
Below average	43 (3.5)	5 (3.7)
Low	6 (0.5)	4 (2.9)

a *P*<.001.

b Due to small group numbers, the categories “nonbinary” and “prefer not to answer” were grouped with “other” responses.

### Top Burdensome Administrative Tasks

**Figure 2.**
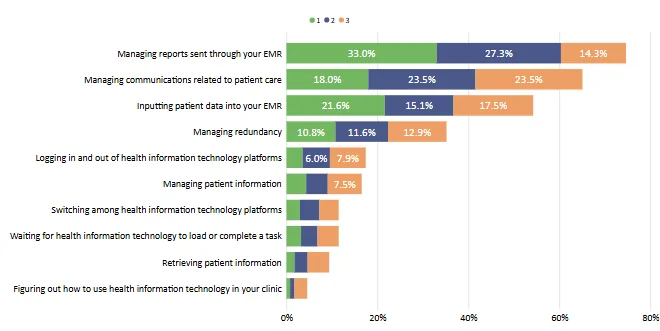
Top 3 most burdensome tasks among Ontario physicians (N=1245).

**Figure 3.**
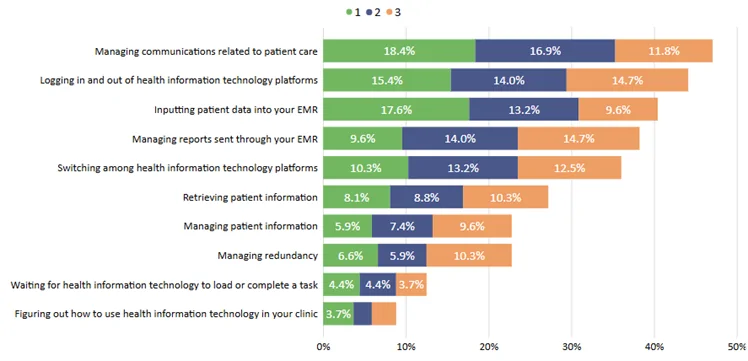
Top 3 most burdensome tasks among Nova Scotia physicians (N=136).

Figures 2 and 3 show the order from most to least burdensome administrative tasks, with bars representing the percentages of the top 3 burdens. Over half of the Ontario sample ranked the following administrative tasks as a top 3 burden: managing reports sent through your EMR (929/1245, 74.6%), managing communications related to patient care (810/1245, 65.1%), and inputting patient data into your EMR (675/1245, 54.2%). The top 3 burdens for the Nova Scotia sample were managing communications related to patient care (n=72, 47.1%), logging in and out of health information technology platforms (60/136, 44.1%), and inputting patient data into your EMR (55/136, 40.4%). For Ontario specialists, the top 3 most burdensome tasks were managing communications related to patient care, inputting patient data into your EMR, and managing reports sent through your EMR. For Nova Scotia specialists, the top 3 most burdensome tasks were logging in and out of HIT platforms, managing communications related to patient care, and retrieving patient information. For both Ontario and Nova Scotia family physicians, the top 3 most burdensome tasks were managing reports sent through your EMR, managing communications related to patient care, and inputting patient data into your EMR.

### Perceptions and Motivations of HIT

Table 3 provides data on perceptions of HIT, as well as motivations for using HIT. For the Ontario sample, significant differences in burnout levels across the agreement categories for certain perception and motivation items are noted. Most Ontario and Nova Scotia physicians agreed with the following beliefs regarding HIT: HIT facilitates coordination of patient care (978/1245, 78.6% in Ontario; 82/136, 60.3% in Nova Scotia) and HIT improves the quality of patient care (819/1245, 65.8% in Ontario; 76/136, 55.9% in Nova Scotia). Most Ontario physicians agreed that HIT makes it convenient for me to access data (861/1245, 69.2%), while over half of Nova Scotia physicians agreed that HIT will revolutionize the future of medicine (82/136, 60.3%). In terms of motivations for using HIT, the most frequently cited reason for using HIT among Ontario physicians was that they either believed HIT would help them provide better patient care or believed HIT would be of value to them, both internally driven sources of motivation. Among Nova Scotia physicians, the most frequently cited reason for using HIT was that they were required to use it, an externally driven source of motivation.

**Table 3.** Perceptions of HIT and motivations for using HIT among physicians.

Survey item and category	Ontario (N=1245), n (%)	Nova Scotia (N=136), n (%)
HIT facilitates communication and coordination of patient care with my administrative staff (if applicable)^a^		
Strongly agree	255 (20.5)	26 (19.1)
Agree	723 (58.1)	56 (41.2)
Neither agree nor disagree	192 (15.4)	32 (23.5)
Disagree	51 (4.1)	17 (12.5)
Strongly disagree	24 (1.9)	5 (3.7)
HIT increases ease of communication with other health professionals^a^		
Strongly agree	143 (11.5)	12 (8.8)
Agree	582 (46.7)	59 (43.4)
Neither agree nor disagree	301 (24.2)	41 (30.1)
Disagree	180 (14.5)	18 (13.2)
Strongly disagree	39 (3.1)	6 (4.4)
HIT improves the quality of patient care^a^		
Strongly agree	152 (12.2)	8 (5.9)
Agree	667 (53.6)	68 (50.0)
Neither agree nor disagree	306 (24.6)	45 (33.1)
Disagree	80 (6.4)	11 (8.1)
Strongly disagree	40 (3.2)	4 (2.9)
HIT is a major reason for my overall job stress^a^		
Strongly agree	250 (20.1)	19 (14.0)
Agree	399 (32.0)	46 (33.8)
Neither agree nor disagree	319 (25.6)	39 (28.7)
Disagree	224 (18.0)	24 (17.6)
Strongly disagree	53 (4.3)	8 (5.9)
HIT makes it convenient for me to access all data I need from different sources for patient care^a^		
Strongly agree	245 (19.7)	14 (10.3)
Agree	616 (49.5)	57 (41.9)
Neither agree nor disagree	163 (13.1)	24 (17.6)
Disagree	160 (12.9)	27 (19.9)
Strongly disagree	61 (4.9)	14 (10.3)
HIT makes the things I want to accomplish easier to get done^a^		
Agree	579 (46.5)	56 (41.2)
Neither agree nor disagree	315 (25.3)	35 (25.7)
Disagree	172 (13.8)	25 (18.4)
Strongly disagree	61 (4.9)	11 (8.1)
HIT negatively impacts my practice^a^		
Strongly agree	100 (8.0)	6 (4.4)
Agree	225 (18.1)	24 (17.6)
Neither agree nor disagree	411 (33.0)	55 (40.4)
Disagree	389 (31.2)	37 (27.2)
Strongly disagree	120 (9.6)	14 (10.3)
HIT prevents me from spending more time with my patients^a^		
Strongly agree	292 (23.5)	14 (10.3)
Agree	370 (29.7)	54 (39.7)
Neither agree nor disagree	269 (21.6)	30 (22.1)
Disagree	259 (20.8)	29 (21.3)
Strongly disagree	55 (4.4)	9 (6.6)
HIT will revolutionize the future of medicine^a^		
Strongly agree	194 (15.6)	14 (10.3)
Agree	488 (39.2)	68 (50.0)
Neither agree nor disagree	370 (29.7)	38 (27.9)
Disagree	145 (11.6)	13 (9.6)
Strongly disagree	48 (3.9)	3 (2.2)
Motivations^b^		
I am required to use HIT at my practice.	673 (54.1)	104 (76.5)
I believed HIT would help me provide better patient care.	867 (69.6)	78 (57.4)
My practice was already using HIT when I joined.	420 (33.7)	57 (41.9)
I believed HIT would be of value to me.	749 (60.2)	51 (37.5)
I believed that the technology would be easy for me to use.	549 (44.1)	41 (30.1)
It would make my practice appear attractive to potential applicants and patients.	186 (14.9)	16 (11.8)
I expected it would be interesting to use HIT at my practice.	187 (15.0)	11 (8.1)

a *P*<.001.

b This was a multiple-response option, so percentages will not add to 100%.

### Physicians’ Typical Work Week

Table 4 shows descriptive statistics on physicians’ work week. Significant associations with technology-related burnout were noted for the Ontario sample. Ontario and Nova Scotia physicians in our sample reported an average work week of 37.4 (SD 14.0) and 39.1 (SD 14.5) hours, respectively, about half of which was spent on direct patient care (50.2%, SD 16.4) in the Ontario sample. Around 26% and 20% of Ontario and Nova Scotia physicians’ time was spent on administration related to patient care. Ontario and Nova Scotia physicians worked an average of 8.6 (SD 7.1) and 4.8 (SD 5.7) additional hours per week, respectively, offsite on administration related to patient care.

**Table 4.** Physicians’ typical work week.

Survey item and category	Ontario (N=1245), mean (SD; range)	Nova Scotia (N=136), mean (SD; range)
Hours worked per week at the practice^a^	37.4 (14.0; 0-90)	39.1 (14.5; 8-80)
Hours worked per week on administration related to patient care^a^	14.0 (9.1; 0-50)	10.5 (9.4; 0-60)
Hours worked per week outside the practice on administration related to patient care^a^	8.6 (7.1; 0-35)	4.8 (5.7; 0-30)
% of time at practice spent on direct patient care	50.2 (16.4; 0-100)	53.0 (28.6; 0-100)
% of time at practice spent on administration tasks related to patient care^a^	25.7 (16.1; 0-100)	25.7 (16.1; 0-100)

a *P*<.001.

### Quality of HIT Support

In terms of HIT support, over 40% in Ontario (539/1245, 43.3%) received support from their technology vendor; over half in Nova Scotia (69/136, 50.7%) reported support from their on-site technical support. In both cases, these forms of support were deemed at least acceptable (75.4% for Ontario and 79.7% for Nova Scotia; Table 5). For the Ontario sample, significant differences by tech-related burnout are noted.

**Table 5.** Sources and quality of support among physicians.

Source of support and quality of support^a^		Ontario, n (%)	Nova Scotia, n (%)
On-site tech support		445 (35.7)	69 (50.7)
Excellent		43 (9.7)	6 (8.7)
Very good		157 (35.3)	14 (20.3)
Acceptable		158 (35.5)	35 (50.7)
Fair		61 (13.7)	11 (15.9)
Poor		26 (5.8)	3 (4.3)
Technology vendor		539 (43.3)	26 (19.1)
Excellent		36 (6.7)	—
Very good		140 (26.0)	6 (23.1)
Acceptable		230 (42.7)	14 (53.8)
Fair		82 (15.2)	4 (15.4)
Poor		51 (9.5)	2 (7.7)
Other		261 (21.0)	41 (30.1)

a *P*<.001.

### Tech-Related Burnout Levels

In the Ontario and Nova Scotia samples, the median level of self-reported tech-related burnout was 7 and 6, respectively; levels with the highest frequency counts were 7 or 8 in the Ontario sample and 5, 6, or 7 in the Nova Scotia sample. A little over half of the Ontario sample reported a tech-related burnout level of at least 7, while almost two-thirds reported a tech-related burnout level of at least 6. In the Nova Scotia sample, most respondents reported a tech-related burnout level of at least 5. Table 6 shows the findings from an additional question on tech-related burnout.

**Table 6.** Percentage of overall work-related burnout due to HIT.

Province	Work-related burnout score, mean (SD; range)
Ontario (N=1245)	42.9 (26.0; 0-100)
Nova Scotia (N=136)	37.0 (25.6; 0-99)

A series of KW rank sum tests on the Ontario data, given its larger sample size, yielded significant results for support quality (KW χ_4_^2^=90.6; *P*<.001), proficiency (KW χ_4_^2^=25.0; *P*<.001), and clinician type (KW χ_1_^2^=21.8; *P*<.001). In this sample, higher quality support and greater proficiency yielded lower levels of self-reported tech-related burnout, whereas lower-quality support and greater (less) proficiency yielded lower (higher) levels of self-reported tech-related burnout. Specialists also tended to report lower tech-related burnout. More unfavorable or favorable perceptions of HIT yielded higher or lower levels of self-reported tech-related burnout. Specifically, agreeing that HIT is the reason for job stress (KW χ_4_^2^=523.98; *P*<.001), prevents patient care (KW χ_4_^2^=305.6; *P*<.001), or negatively impacts their practice (KW χ_4_^2^=360.8; *P*<.001) yielded significantly higher levels of self-reported tech-related burnout. Conversely, all other items yielded statistically significant differences in median self-reported tech-related burnout, with more favorable views of perception linked to lower levels of self-reported tech-related burnout. Pearson product moment correlation tests also yielded a significant correlation between the overall score on perceptions and tech-related burnout (*r*=−0.56; *P*<.001). Hours per week at practice (*r*=0.19; *P*<.001), hours per week (*r*=0.33; *P*<.001) or percent of time (*r*=0.21; *P*<.001) spent on administration related to patient care, and extra hours per week spent on administration related to patient care (*r*=0.34; *P*<.001) yielded weak-to-moderate positive correlations with self-reported tech-related burnout.

Systematic stepwise regression tests yielded a robust, well-fitting model explaining about 60% of the variation in self-reported tech-related burnout (*R*^2^=0.60; *F*(7,1237)=266.515; *P*<.001). Table 7 shows the performance of individual factors in the regression model. Percentage burnout tech (ie, the respondent’s estimated percentage that technology contributes to their burnout) was subsequently removed due to difficulties in interpretation, which resulted in a model accounting for about half of the variation in self-reported tech-related burnout, with 6 stable, meaningful, and potentially actionable factors. All ordinal factors evidenced monotonic, near-linear trends. Follow-up statistical tests were conducted to confirm the regression model. Neither multicollinearity (ie, through examination of variance inflation factors) nor autocorrelation among residuals (ie, through the Durbin-Watson test) was observed. A Bonferroni test for outliers indicated no studentized outliers (*P*<.05).

**Table 7.** Regression factor coefficients.

Predictor	Coefficients (SE)	*t* test (*df*)^a^	*P* value
Intercept	1.841 (0.333)	5.534 (138)	<.001
Communication	–0.202 (0.050)	–4.021 (138)	<.001
Prevent patient care	0.227 (0.048)	4.695 (138)	<.001
Reason job stress	0.661 (0.059)	11.277 (138)	<.001
Support quality	–0.194 (0.046)	–4.186 (138)	<.001
Hours per week	0.017 (0.003)	4.903 (138)	<.001
Hours per week extra	0.052 (0.007)	7.529 (138)	<.001

a Two-tailed *t* test.

### Qualitative Findings

Both coding teams began by assigning responses to the main qualitative question, “How does health information technology contribute to or help to alleviate your burnout?” into 2 themes: negative (corresponding to “contributes to”) and positive (corresponding to “alleviates”). With the larger Ontario sample, QSR NVivo software was used to facilitate coding, and automated sentiment analysis was attempted but was found to be inaccurate upon review—with frequent misattributions of sentiment due to expressive ambiguity or sarcasm, for example. As described in the “Methods” section, blank or difficult-to-interpret responses were excluded from coding.

Using this approach, 146 references were coded as negative and 31 as positive in the Nova Scotia sample, with 864 coded as negative and 657 as positive in the Ontario sample. Each coding team then proceeded with open coding for themes that emerged under each of the 2 larger categories. Codebooks were developed for each province’s sample with codenames and definitions. Once this step was completed, the 2 coding teams compared codebooks to identify similarities across themes—with a focus on aligning code definitions rather than codenames.

From this we discovered that the 2 most common positive themes in both groups were aligned, although in reverse order. The first of these in the Ontario sample dealt with HIT’s capacity to facilitate data management and accessibility (coded as “data management”—168 references; in the Nova Scotia sample coded as “accessibility”—7 references, the second most common). The second refers to administrative efficiency (coded as “regular task completion”—112 references; in the Nova Scotia sample coded as “efficiency”—11 references, the most common).

The considerably larger set of references for the negative statements also offered greater complexity and some differences between the samples. The highest frequency of negative statements in the Ontario sample referred to report management. The second most common theme in the Ontario sample and the most common in the Nova Scotia sample were aligned: the challenges of systems that “don’t talk to each other,” that is, do not interact easily (some variation of which we saw frequently), so that accessing all the necessary patient data requires logging in and out of multiple unconnected platforms. These responses often cited the interruptive nature of recalling different login information and completing dual-factor authentication. These types of references were initially coded as “interoperability and integration” in the Ontario sample and “fragmented” in the Nova Scotia sample.

Otherwise, there were additional differences in the remaining negative codes. Table 8 shows the 4 most common negative subthemes coded in each sample, including the codename, definition, number of references for each code, and a sample quote.

**Table 8.** Qualitative coding and sample responses: negative experiences.

Code	Definition	Count, n	Sample quote
Ontario (n=684)			
Managing reports	Duplicate, redundant, irrelevant, too long, labeled incorrectly, or poorly formatted reports.	177	“I have to read all reports that come in, even the useless ones. Waste of my time.”
Interoperability and integration	Systems don’t speak to one another; cognitive overload from switching between platforms and authentication modes.	120	“Lots of platforms promising to improve my workflow, but each requires a unique login and password.”
Work-life balance	EMR availability 24/7 leads to expectation to access beyond work hours.	111	"Never get a break from checking, even when on vacation.”
Referrals	Too many different forms, and specialists reject if forms are not filled correctly; no centralized system to pull information.	102	"Different institutions have different referral platforms and logins.”
Nova Scotia (n=146)			
Interoperability and integration	Fragmented systems require logging in and out of each system and systems do not communicate between one another.	30	"You need to search multiple different techs to find all the data needed.”
Accessibility	Data cannot be found; uploading delays; stored in the wrong place; difficulty navigating.	17	“Limited access to some information locations or difficulty finding specific documentation.”
Time	Time-consuming, slower to use HIT than paper.	17	“Extra hours for admin tasks leave me tired and use up brain energy.”
Usability	HIT systems are not user-friendly, not intuitive, too difficult to use, and technologically challenging.	17	“Implementation with our EMR was so non-user-friendly that we had to deimplement it and revert to phone calls.”

## Discussion

### Interpretation

Despite notable differences between the provinces in HIT maturity, complexity, adoption rates, practice models, and physician characteristics, common themes emerged surrounding HIT involvement in burnout. While physicians identified many positive aspects of HIT, our findings show that most physicians self-assessed their tech-related burnout level as high: 6 out of 10 in Nova Scotia and 7 out of 10 in Ontario. On average, Ontario respondents reported that 43% of their overall burnout is tech-related, while Nova Scotia respondents reported 37%. Ontario respondents reported greater tech-related burnout and attributed a higher percentage of their overall burnout to technology. Over a third of burnout in our respondents’ self-reports is attributable to HIT.

The top-ranked administrative burdens share commonalities across provinces. Nova Scotia respondents identified managing communications as their primary burden; in Ontario, this ranked second after managing reports due to the effects of nonstandardized implementation of Health Report Manager (HRM) in hospitals (a long-standing issue, which is in the process of being addressed in the province). Both samples ranked inputting patient data into their EMR in third place and logging in and out of technology platforms in their top 5 (second in Nova Scotia and fifth in Ontario). This issue emerged strongly in the qualitative material, which revealed frustrations with logging in and out of non-EMR-integrated platforms for referrals, data portals, etc, which all require unique logins, passwords, and dual-factor authentication. Responses suggest that physicians experience these as both adding to cognitive burden and taking time away from patient care. In both samples, ongoing change initiatives—the provincial rollout of OPOR in Nova Scotia and both HRM improvement initiatives [28] and the Primary Care Action Plan in Ontario [29]—are likely to have an impact on the future experience of administrative burdens and perhaps shift these rankings. Other initiatives at the national level to improve vendor attention to interoperability and standards for HIT solutions brought to market may also have an impact on the physician experience. Bill S-5, the *Connected Care for Canadians Act,* would require HIT vendors to make the technology they sell to clinics interoperable, with penalties for “data blocking.” [30-32] Further, a national effort to standardize data content nationwide is underway with the Pan-Canadian Health Data Content Framework [33]. In the meantime, expensive HIT tools are outpacing our health system’s capacity to adapt. For example, auto-population of forms, patient data integration, and AI assistance are not uniformly regulated or implemented across Canadian practices and provinces [34].

One of the strengths of our approach is the elicitation of candid physician comments via the open text survey questions and our subsequent analysis. This has allowed us to see more layers of the physician experience, especially the complex “love/hate” relationship physicians have with their HIT. In the qualitative material, we found commonality across the Ontario and Nova Scotia samples in terms of the expressed positives of HIT, particularly for data management and administrative efficiency. These align with the reasons HIT was developed and implemented, particularly where government investment is involved. We note with interest that while both samples showed common issues with interoperability and integration (in line with the quantitative results), there were differences in how respondents expressed their other challenges with HIT. In the Ontario sample, we found high counts of references to specific issues, for example, incoming document management and referrals; this was not the case in the Nova Scotia sample, where “usability” was a common theme. This may be due to differences in the samples (ie, higher counts of specific mentions in the larger Ontario sample). We also suggest that the sample from Ontario’s more mature HIT environment may have progressed beyond general usability concerns to complaints about more specific challenges.

Perceptions about HIT showed some agreement with positive statements about technology. This tended to be stronger in Ontario, perhaps due to that province being a more seasoned HIT landscape than Nova Scotia and some anticipatory concern in Nova Scotia about the province’s upcoming move from a largely paper-based system to OPOR. In both samples, more than half of respondents agreed with positive statements about the impact of HIT on patient care and data access. However, about half of each sample also strongly agreed that HIT prevents spending more time with patients. Indeed, both samples reported spending about half their time on direct patient care, with a notable proportion on administration (approximately 20% in Nova Scotia and 25% in Ontario). These particular (types of) perceptions are conceptually similar to tech-related burnout; therefore, one would expect robust associations. However, findings were moderate and somewhat unclear, which may indicate that perceptions are mediated or moderated by other yet unstudied attributes. Many open text responses contained some version of “I spend more time with the computer than with my patients” related to documentation and accountability expectations that increase screen-related tasks and dominate the patient encounter. These negative perceptions associated with administrative burdens compete with the perceived benefits to patient care. This aligns with findings elsewhere regarding the contribution of daily frustrations, task, and workload burden to burnout [4,7] and underscores many physicians’ complex relationships with HIT.

HIT is (or should be) a highly modifiable contributor to burnout; improving physicians’ experience of HIT could decrease some burnout overall. Physicians may see the benefits of HIT for patient care in principle; however, a physician’s ability to yield optimal value from HIT depends on usability, ease of switching between platforms and nonstandardized forms and referrals, and lessening administrative burden, all of which influence time spent on patient care. HIT co-designed with physicians [35] could lead to interoperability and usability standards that better address the needs of a variety of different practice types, physician characteristics, and proficiency levels—with positive effects system-wide. A focus on human factors in the design of HIT can offset the usability challenges that may be unintentionally introduced by technology vendors in disregarding the realities of clinical practice [36-38]. An example of co-design supporting interoperability exists in the Belize Health Information System implementation, where Canadian and Belizean health professionals worked with community input for design and deployment [39,40]. The result was a system that was easy to use and reduced administrative tasks, including searches and redundancies—while also generating low development and maintenance costs and improving patient outcomes. Successful implementation of HIT relies on the involvement of end-user health care professionals at the outset to better translate the realities of clinical practice workflow to technological solutions.

### Future Directions

The OPOR initiative presents an opportunity for a prestudy and poststudy in the Nova Scotia context, similar to other preimplementation or postimplementation studies in this area—particularly to investigate the impact on dedicated patient care time [41]. In Ontario, government-led initiatives such as the Primary Care Action Plan [29] intend to address several areas of HIT that contribute to administrative burden; plans to standardize implementation of HRM in hospitals will also address a large contributor to unwieldy inboxes [28]. A follow-up questionnaire pursuant to implemented changes could explore whether these have had an impact on administrative burden and burnout. There is also an opportunity to further investigate differences between specialists and primary care physicians. While the Nova Scotia sample had a higher representation of specialists than the Ontario sample, the area of specialty was primarily pediatrics, where physicians often act as de facto family physicians, providing incremental care over years to the same families. As we did find lower levels of self-reported tech burnout among specialists compared to family physicians, capturing the difference between “true” specialists (those who provide secondary rather than primary care) and family physicians may provide insights from the greater contrast in practice style. Although there are similarities across specialists and family physicians in terms of most burdensome tasks, the qualitative analysis in our study suggests that the reality of administrative burden in clinical practice may differ between the 2 groups. In turn, this may be contributing to tensions (eg, primary care physicians express frustration with nonstandardized referral forms and the extra administrative burden these forms generate).

### Limitations

These findings are preliminary and exploratory and provide a foundation on which to further explore tech-related burnout. We recognize as a limitation that our measure is subjective, based on self-report, and not a validated objective measure. These findings are based on perceptions and associations between self-reported items. Because we used multiple avenues to promote the questionnaire, respondents were not prevented from accessing the questionnaire more than once; however, we controlled for duplicate responses via unique identifiers. Because we used various sampling methods and exclusion criteria, it is difficult to discern the sampling frame and calculate response rates. The sample is unlikely to be representative. There are limitations to generalizability associated with the small Nova Scotia sample and lack of variability in characteristics that preclude certain statistical tests, hinder precision, and potentially introduce bias, including overestimation of effect sizes. Certain response biases (eg, respondents with higher levels of burnout may have been more motivated to participate), common in this type of research, may have influenced the analysis. We attempted to address related biases in our analysis and reporting. Because findings primarily reflect physician experiences within 2 Canadian provincial health systems, they may not reflect or represent experiences in other health care settings. Although measured on a scale following best practices and adapted from similar scales noted in the literature, our tech-related burnout scale has not yet been validated and is subject to interpretation. Although yielding a significant predictive model, it is difficult to ascertain what an increase of a burnout level of “5” to “6” entails clinically. Qualitative research may help to describe these types of scales. The inductive qualitative coding approach, in which we created separate codebooks for Ontario and Nova Scotia, posed limitations to the comparability of that coding. Further, thematic coding may be subject to bias due to the small number of coders involved (2 for Ontario, 1 for Nova Scotia). Furthermore, this analysis should be viewed as exploratory as we continue to understand tech-related burnout and further refine our approach. We note evidence of validity via the strong correlation yielded between our burnout scale and “Percent burnout from technology.” Furthermore, some of the regression factors were measured on scales despite evidencing monotonic, near-linear trends from Bayesian generalized nonlinear multilevel modeling tests. Finally, self-report questionnaires are subjective, so we did not secure objective measures of either burnout or time usage. We followed best practice approaches such as item checks of respondents’ attention and piloted ease of questionnaire completion. Our findings are best interpreted as reflecting respondent perceptions and experiences.

### Conclusion

Our study provides a broad picture of the physician experience of HIT in the 2 settings we studied. Physician needs may change depending on their location, practice type, specialty, scope of practice, and day-to-day schedule. HIT should have the flexibility to accommodate these needs and be tailored to the people it serves. For this to happen, physicians need to be deeply involved, including in the design of programs to simplify clinical workflows, for example, the use of AI in clinical decision support. Physicians expressed a need to spend more time with patients than with HIT. The current plethora of largely unregulated vendors for an increasingly diverse set of platforms to address the needs of clinical practice, and the lack of full patient-centered integration, may result in high costs for less user-friendly systems. This study reinforces the complexity of HIT systems and their impact on administrative burden. Ongoing involvement of physicians—who actively use these systems—in HIT design and implementation is essential.

This study contributes practical relevance to the growing literature examining the relationship between technology and burnout [7,8,42-45] with analysis and interpretation of physician perspectives on their complex relationships with HIT. HIT may facilitate patient data collection, management, and use, but it can also add cognitive and administrative burdens to physicians’ everyday work life. HIT is not inherently protective or harmful; rather, physician experience depends heavily on usability, interoperability, workflow alignment, and meaningful physician involvement in design and implementation to address their clinical realities.

## Supplementary material

10.2196/93266 Multimedia Appendix 1 Survey instrument.

## Abbreviations

CMA: Canadian Medical Association
DNS: Doctors Nova Scotia
FOM: Faculty of Medicine
HIS: hospital information system
HIT: Health information technology
HRM: Health Report Manager
KW: Kruskal-Wallis
NSH: Nova Scotia Health
OMA: Ontario Medical Association
OMD: OntarioMD
OPOR: One Person One Record
