# Supplementary material for: Physicians’ Complex Relationships With Health Information Technology and Burnout: Cross-Sectional Survey Study
Source: JMIR Hum Factors. 2026 Sep 23;13:e93266. doi: 10.2196/93266 (PMC13600587; doi:10.2196/93266)
Supplement: Supplementary file 1 [file humanfactors-v13-e93266-s001.docx]

# Appendix: Survey Instrument

**RECRUITMENT (Ontario)**

Subject line: A message on tech-related burnout from OMD's CMO, Dr. Chandi Chandrasena

Dear {{contact.firstname}} {{contact.lastname}},

As a physician, I share many of the challenges I’ve heard you communicate to OntarioMD through your messages to me, our conferences, and our surveys. I understand the pressures you face.

You may be wondering why we’re taking more of your precious time. Why should you take 5-10 minutes to respond to yet another survey? The answer is that these survey questions are designed to get at the details about the technology you use in your practice. We want to know **which tools make your life easier, and which do the opposite.** The reason it’s so important to get your responses to these questions is that we’re analyzing them for patterns in practice type, location, size, etc. so we can get the information we need to deliver the support YOU need.

**Your responses matter – they help us understand how to serve you better and what to offer you in the future.** In the past, your responses to our surveys contributed to our initiation of projects such as the HRM Task Force and AI Scribe.

**Please complete the survey by March 15, 2024.**

[CLICK HERE TO START SURVEY](https://ncv.microsoft.com/y0L8Z0ttvZ)

‌Your answers will be kept confidential, and anything we share (e.g., with stakeholders) will be in an aggregated and de-identified format. All personal information is collected, used, and disclosed in accordance with OMD's [Privacy Policy (ontariomd.ca)](https://www.ontariomd.ca/privacy-policy). We do not share or sell your personal information to third parties.

If you have any questions, then please contact [support@ontariomd.com](http://support@ontariomd.com/). Thank you for your time and we look forward to your feedback!

Dr. Chandi Chandrasena,  MD, CCFP, FCFP

Chief Medical Officer, OMD

**CONSENT ACKNOWLEDGEMENT (Nova Scotia)**

1. You are invited to take part in a research study being conducted by Dara Liu, an undergraduate medical student, and Dr. Jeannette Comeau and Dr. Janice Graham, faculty members at Dalhousie University, Department of Pediatrics, Faculty of Medicine, in collaboration with OntarioMD.

**Purpose:** The purpose of this research is to investigate the relationship between health information technology (HIT) and the burnout experienced by physicians in Nova Scotia and Ontario. HIT is any technology that is used to input, use, extract, or analyze information within healthcare (e.g., electronic medical records, electronic systems for documenting encounters, logs for clinic letters, getting diagnostic tests, making referrals, etc.). By surveying physicians on use of technology during patient care, we hope to uncover the ways in which technology contributes to burnout and avenues for improvement.

**Your Role:** If you choose to participate in this research, you will be asked to complete a short, 20 item, online questionnaire about the technology you use for patient care, its functionality, your level of burnout associated with the technology, and some demographic characteristics. The survey should take under 10 minutes to complete.

**Privacy & Risks:** The only identifying information collected will be the postal code of your primary practice location. There are no questions that ask for identifying details such as your name or email address. Your participation in this research is entirely your choice. You may stop the survey at any time if you no longer want to participate; all you need to do is close your browser before submitting your responses. Incomplete surveys will not be included in the analyses. If you do complete your survey and you change your mind later, the information you provided will be included because the data is anonymous and cannot be linked to you. There is no identifying information tied to your survey responses. All responses will be saved on a secure, encrypted server hosted by OntarioMD. Only the research teams at Dalhousie University and OntarioMD will have access to the results. The minimal risks associated with this study are no greater than those you encounter in your everyday life. If you feel any discomfort or are concerned about the content of this survey, please contact Dara Liu (see below).

**Benefits:** There will be no direct benefit to you in participating in this research. The research, however, might contribute to new knowledge on the landscape and relationship of technology used for patient care and physicians in Nova Scotia. If you would like to see how your information is used, please free to contact Dara Liu (see below).

**Outcomes:** I will describe and share general findings of this research in conference presentations and publications. I will keep the anonymous survey information indefinitely as it may be used in future research.

**Questions:** You can discuss any questions you have about this study with Dara Liu and Dr. Janice Graham at [dara.liu@dal.ca](mailto:dara.liu@dal.ca). Please ask as many questions as you like before or after participating. If you have any ethical concerns about your participation, you may contact Research Ethics, Dalhousie University at (902) 494-3423, or email [ethics@dal.ca](mailto:ethics@dal.ca) (and reference REB file # 2023-6993).”

**To thank you for your participation, you can choose to enter your name into a draw for 1 of 4, $50 Amazon gift cards!**

If you wish to participate please click below, otherwise close this window.

- I have read the above information and agree to participate in this survey [Skip to “About Your Health information Technology”]

**CONSENT ACKNOWLEDGEMENT (Ontario)**

1. All personal information is collected, used and disclosed in accordance with the OntarioMD Privacy Policy (www.ontariomd.ca/privacy-policy). You have received this because you are already on our contact list. We do not rent or sell your personal information to third parties, and you can unsubscribe at any time. If you wish to participate please click below otherwise close this window.

- I have read the above information and agree to participate in this survey [Skip to “About Your Health information Technology”]

**ABOUT YOUR HEALTH INFORMATION TECHNOLOGY**

By *health information technology* we mean any technology that is used to support patient care in your primary practice location (e.g., electronic systems for documenting encounters, getting diagnostic tests, making referrals, etc.)

2. Do you currently use health information technology in your primary practice location? Select one. [only one response accepted]

- Yes, exclusively [Skip to Question 4]
- Yes, in combination with paper documentation [Skip to Question 3]
- No [Skip to ‘About You’]

3. Approximately what percent of the time do you use paper as opposed to health information technology to support patient care? Please enter a number. [response must be a number]

4. How would you rate your proficiency in using health information technology to support patient care? By proficiency, we mean your knowledge, skills, and comfort level. Select one. [only one response accepted]

- High
- Above average
- Average
- Below average
- Low

5. If you had a problem with health information technology at your practice, to whom would you go **first** for support? Select one. [only one response accepted]

- The technology vendor
- Onsite tech support
- Other: ____________________

6. Using the scale below, please rate the quality of your typical health information technology support. Select one. [only one response accepted]

- Excellent
- Very good
- Acceptable
- Fair
- Poor

7. Why do you use health information technology to support patient care at your primary practice location? Select all that apply. [multiple responses accepted]

- I am required to use health information technology at my practice.
- I expected it would be interesting to use health information technology at my practice.
- I believed health information technology would be of value to me.
- I believed that health information technology would help me provide better patient care.
- It would make my practice appear attractive to potential applicants and patients.
- I believed that the technology would be easy for me to use.
- My practice was already using health information technology when I joined.
- Other: ____________________

8. To what extent do you agree or disagree with the following statements? Select one. [only one response accepted]

“**Health information technology**…

| Statement | Strongly Agree | Agree | Neither Agree nor Disagree | Disagree | Strongly Disagree |
| --- | --- | --- | --- | --- | --- |
| is a major reason for my overall job stress.” |  |  |  |  |  |
| makes the things I want to accomplish easier to get done.” |  |  |  |  |  |
| will revolutionize the future of medicine.” |  |  |  |  |  |
| prevents me from spending more time with my patients.” |  |  |  |  |  |
| increases ease of communication with other health professionals.” |  |  |  |  |  |
| negatively impacts my practice.” |  |  |  |  |  |
| improves the quality of patient care.” |  |  |  |  |  |
| facilitates communication and coordination of patient care with my administrative staff (if applicable).” |  |  |  |  |  |
| makes it convenient for me to access all data I need from different sources for patient care.” |  |  |  |  |  |

**ABOUT YOUR AVERAGE WORK WEEK**

9. In an average week, how many hours do you work at your primary practice location? Please enter a number.

__________ hours [response must be a number]

10. Approximately what percent of your regular working hours per week at your primary practice location is spent on direct patient care? Please enter a number.

__________ % [response must be a number]

11. Approximately what percent of your regular working hours per week at your primary practice location is spent on administration related to patient care? Please enter a number.

__________ % [response must be a number]

12. On average, how many hours per week do you spend on administration related to patient care? Please enter a number.

__________ hours per week [response must be a number]

13. On average, how many extra hours do you work per week outside your primary practice location on administration related to patient care? Please enter a number.

__________ hours per week [response must be a number]

14. Please rank the following 10 administrative tasks in order from greatest burden to least, by clicking and dragging each task higher or lower as desired:

- Managing communications related to patient care
- Managing reports sent through your EMR
- Inputting patient data into your EMR
- Logging in and out of health information technology platforms
- Switching among health information technology platforms
- Figuring out how to use health information technology in your clinic
- Managing redundancy (e.g., duplicate reports sent from various sources, inputting the same information in more than one place, etc.)
- Retrieving patient information
- Managing patient information
- Waiting for health information technology to load or complete a task

**ABOUT YOUR TECH-RELATED BURNOUT**

15. On a scale of 0 to 10 where 0 is not at all burned out and 10 is extremely burned out, how would you rate your level of burnout associated with health information technology? Select one. [only one response accepted]

0	1	2	3	4	5	6	7	8	9	10

16. Approximately what percent of your overall work-related burnout is due to health information technology? Please enter a number.

__________ % [response must be a number]

17. What other things burn you out at work?

______________________________________________________________________________________________________________________________________________________________________________________________________________________________________________________________________________________________________

18. Please tell us how health information technology contributes to or helps to alleviate your burnout

______________________________________________________________________________________________________________________________________________________________________________________________________________________________________________________________________________________________________

**ABOUT YOU**

We ask for the below identifying information in order to categorize and group responses, as well as identify trends within these attributes. All personal information is collected, used and disclosed in accordance with the OntarioMD Privacy Policy ([www.ontariomd.ca/privacy-policy](http://www.ontariomd.ca/privacy-policy)). We do not share your data with any organization. All data will be kept secure and confidential. In analysis, data will be de-identified and aggregated.

19. What is your CPSO/CNO number?

__________ [response must be a number]

20. Which of the following genders do you most identify with? Select one. [only one response accepted]

- Male
- Female
- Transgender Male
- Transgender Female
- Non-binary
- Prefer not to answer
- Other: ____________________

21. What is your age? Please enter a number.

__________ years [response must be a number]

22. People are often described by racialized terms that may influence the way we are treated by individuals and institutions, and this may affect our health. Which category best describes you? Select one. If you identify as mixed race then please select all that apply.

- Black (e.g., African, African Canadian, Afro-Caribbean descent)
- East Asian (e.g., Chinese, Japanese, Korean, Taiwanese descent)
- Indigenous (e.g., First Nations, Inuk/Inuit, Métis) [if selected then go to #16 else go to #17]
- Latin American (e.g., Hispanic or Latin American descent)
- Middle Eastern (e.g., Arab, Persian, West Asian descent {e.g., Afghan, Egyptian, Iranian, Kurdish, Lebanese, Turkish})
- South Asian (e.g., Bangladeshi, Indian, Indo-Caribbean, Pakistani, Sri Lankan)
- Southeast Asian (e.g., Cambodian, Filipino, Indonesian, Thai, Vietnamese)
- White (e.g., European descent)
- Prefer not to answer
- Another racialized category — please specify: ____________________

23. Do you identify as First Nations, Inuk/Inuit and/or Métis? Select all that apply. [multiple responses accepted]

- Yes, First Nations
- Yes, Inuk/Inuit
- Yes, Métis
- Yes, other — please specify: ____________________
- Do not know
- Prefer not to answer

24. How many years have you been practicing medicine? Please enter a number.

__________ years [response must be a number]

25. Which of the following best describe(s) you? Select one. [only one response accepted]

- Family Physician
- Specialist
- Resident
- Nurse
- Nurse practitioner
- Clinic manager
- Other: ____________________

**ABOUT YOUR PRACTICE**

We ask for the below identifying information in order to categorize and group responses, as well as identify trends within these attributes. All personal information is collected, used and disclosed in accordance with the OntarioMD Privacy Policy ([www.ontariomd.ca/privacy-policy](http://www.ontariomd.ca/privacy-policy)). We do not share your data with any organization. All data will be kept secure and confidential. In analysis, data will be de-identified and aggregated.

26. What is your primary practice type? Select one. [only one response accepted]

- Group practice
- Solo practice
- Hospital based practice
- Other: ____________________

27. In the last year, which of the following payment methods accounted for most of your professional income? Select one. [only one response accepted]

- Fee-for-service
- Salaried
- Clinical/academic funding plan
- Other: ____________________

28. What is the postal code at your primary practice location?

__________ [response must be a postal code with no spaces]

29. If you would like to be included in the draw for one of four $50 Amazon gift cards then please enter your email address below so that we may contact you in case you win a gift card. **(Nova Scotia)**

__________ [response must be an email address]
